# Comparative Study of Polyethylene Films Embedded with Oxide Nanoparticles of Granulated and Free-Standing Nature

**DOI:** 10.3390/polym14132629

**Published:** 2022-06-28

**Authors:** Stavros Christopoulos, Nicos C. Angastiniotis, Valerie Laux - Le Guyon, Eliane Bsaibess, Loukas Koutsokeras, Benoît Duponchel, Joumana El-Rifai, Liang Li, Ahmed Slimani

**Affiliations:** 1 Department of Sciences and Engineering, Sorbonne University Abu Dhabi, Abu Dhabi 38044, United Arab Emirates; valerie.leguyon@sorbonne.ae (V.L.-L.G.); eliane.bsaibess@sorbonne.ae (E.B.); joumana.elrifai@sorbonne.ae (J.E.-R.); liang.li@sorbonne.ae (L.L.); ahmed.slimani@sorbonne.ae (A.S.); 2 Department of Mechanical Engineering and Materials Science and Engineering, Cyprus University of Technology, Limassol 3041, Cyprus; l.koutsokeras@cut.ac.cy (L.K.); 3 Unit of Dynamics and Structure of Molecular Materials, University of Littoral Opal Coast, 59140 Dunkerque, France; benoit.duponchel@univ-littoral.fr (B.D.)

**Keywords:** nanoparticles, metal oxides, nanopowders, polymer nanocomposites, optical films, low-density polyethylene

## Abstract

Nanocomposite polymer films are a very diverse research field due to their many applications. The search for low-cost, versatile methods, producing regulated properties of the final products, has thus become extremely relevant. We have previously reported a bulk-scale process, dispersing granulated metal oxide nanoparticles, of both unary and multi-component nature, in a low-density polyethylene (LDPE) polymer matrix, establishing a reference in the produced films’ optical properties, due to the high degree of homogeneity and preservation of the primary particle size allowed by this method. In this work, unmodified, free-standing particles, namely zinc oxide (ZnO), titanium dioxide ($TiO_{2}$), aluminum oxide ($Al_{2}O_{3}$), and silicon dioxide ($SiO_{2}$) are blended directly with LDPE, and the optical properties of the fabricated films are compared to those of films made using the granulation process. The direct blending process evidently allows for control of the secondary particle size and ensures a homogeneous dispersion of the particles, albeit to a lesser extent than the granulation process. Despite the secondary particle size being comparatively larger than its granulated counterpart, the process still provides a regulated degree of deagglomeration of the free-standing oxide particles, so it can be used as a low-cost alternative. The regulation of the secondary particle size tunes the transmission and reflection spectra, in both unary and mixed oxide compositions. Finally, the direct blending process exhibits a clear ability to tune the energy band gap in mixed oxides.

## 1. Introduction

Advances in nanotechnology have led to the development of new fabrication techniques incorporating nanomaterials of different shapes and sizes embedded in bulk polymer hosts [1,2,3,4,5,6,7]. The synergy results between particles and matrix have been found to have profound effects on the end products’ optical properties [8,9,10,11,12]. More importantly, controlling the main parameters of the fabrication process, such as the size, shape, concentration, and spatial distribution of the dispersoid, as well as its blending into the polymer matrix, allows a direct regulation of the materials’ properties [13,14,15,16,17].

Within this framework, in previously published work [18], bulk quantities of unary and mixed deagglomerated oxide particles of predetermined composition were homogeneously dispersed in low-density polyethylene (LDPE) films. A multi-stage granulation process was designed to establish the utmost uniform oxide dispersion in the film while regulating the primary and secondary size of the particles. Given the homogeneous dispersion, the capability to control both the primary and secondary particle size of the oxides provides the means for a tailored optical performance. In particular, it was shown that by using predetermined amounts of mixed granulated oxides, a film could be fabricated with a made-to-order optical performance, characterized by a predefined energy band gap and transmission fraction value. The latter could be adjusted anywhere in between the transmission fraction values of its corresponding pure oxides when used on an individual basis.

Even though the tailored optical performance of the LDPE film is of unequivocal value and the applications are numerous, its realization is dependent on the primary and secondary oxide particle sizes that are obtained through a granulation process that might be prohibitive in cost.

Hence, this work reports results on an alternative approach using the same unary and mixed unmodified, free-standing oxide particles. However, in this case, the dispersion is achieved by blending them directly with LDPE. Despite its limitation in achieving a high degree of homogeneity and its inability to control the primary particle size, this process provides a regulated, albeit limited, deagglomeration capability of the free-standing oxide particles.

This technique can be used as a low-cost alternative for controlling the transmission of the films, especially when compared directly to the standardized transmission of films with granulated particles. More specifically, the LDPE films fabricated by direct mixing of the oxides exhibit lower transmission than their granulated counterparts, due to their larger secondary particle size. Hence, a comparative study between the two techniques allows for a generalized and direct evaluation of the optical performance of the films produced by the direct mixing of free-standing particles, opening up new methods for improvement.

## 2. Sample Preparation and Characterization

### 2.1. Materials

A bulk-scale process is implemented for the production of nanostructured film composites comprising single and mixed oxide particle formulations dispersed in an LDPE matrix. The choice of LDPE as a hosting material for the dispersed nanoparticles was based on its optical properties allowing for a wide range of applications [19,20,21,22], its low cost, and its compatibility with established fabrication processes. As shown in Table 1, four types of free-standing metal oxides, namely ZnO (Sigma-Aldrich, Darmstadt, Germany), $SiO_{2}$ (Sigma-Aldrich and Alfa Aesar, Karlsruhe, Germany), $TiO_{2}$ (Sigma-Aldrich), and $Al_{2}O_{3}$ (Alfa Aesar, SSNano, Houston, TX, USA and Sigma-Aldrich) were embedded in LDPE, either in single or mixed formulation. The embedded single-oxide formulations are classified into three categories, depending on their free-standing particle size range: (i) less than 100 nm, (ii) between 0.1 and 1 μm, and (iii) between 1 and 10 μm.

Four single oxide formulations (i.e., $ZnO,TiO_{2},SiO_{2}$, and $Al_{2}O_{3}$) of all sizes, and three 1:1 equimolar mixed oxide formulations, where each formulation comprised a mixture of two single oxides (i.e., $TiO_{2}$-$SiO_{2},Al_{2}O_{3}$-$SiO_{2}$, and $TiO_{2}$-$Al_{2}O_{3}$) were used to produce twelve films of free-standing particles, embedded in LDPE. The aforementioned single oxides, with the exception of ZnO, were used in unary and mixed composition to produce granulated particles, which were subsequently embedded in the LDPE matrix, producing six additional films. Finally, a film of neat LDPE was fabricated for reference.

### 2.2. Free-Standing Single and Mixed Oxides

Each type of free-standing oxide formulation was blended with LDPE through an extrusion pelletizing process [23]. This was implemented by an AXON BX-18 bench extruder that was set up for pelletizing, producing 13 masterbatches, including one of neat LDPE. The masterbatches contained the active ingredient (i.e., as defined by the type of oxide formulation) in a predetermined proportion, which was set at 20% by mass (20 g of active ingredient blended with 80 g of LDPE). The three mixed-oxide masterbatches were fabricated using the same composition, by blending 20% by mass 1:1 equimolar mixtures of two single oxides (i.e., $TiO_{2}$-$SiO_{2},Al_{2}O_{3}$-$SiO_{2}$, and $TiO_{2}$-$Al_{2}O_{3}$), as given in Table 2. It is noted that the oxides used for this purpose belong to the smallest free-standing particle range (< 100 nm) of Table 1.

Each masterbatch was subsequently blended at 10% by mass with LDPE, through an extrusion film-blowing process [24] that was implemented by the aforementioned AXON BX-18 bench extruder, which was set up with a film-blowing head. As the masterbatch proportion in the film was set at 10% (i.e., 100 g of masterbatch in 900 g of LDPE), the active ingredient in the film was reduced to 2% by mass (i.e., 20 g of the dispersoid in 980 g of LDPE). It is finally noted that the films have a thickness of 70 μm.

### 2.3. Granulated Single and Mixed Oxides

Three of the as-received oxide formulations, as shown in Table 1, were suspended, on an individual basis, in water by using poly(acrylic acid) dispersant (PAA). The oxides used for this purpose belong to the particle range < 100 nm. The as-suspended particles were deagglomerated by applying ball and/or planetary milling and then were wet-sieved at 45 μm before being forced through a nozzle in the presence of liquid nitrogen to yield granulated particles (granules) in the range of 20–300 μm that were subsequently freeze-dried in vacuum at 1.5 mbar and −16 °C.

The mixed oxide formulations (i.e., $TiO_{2}$-$SiO_{2},Al_{2}O_{3}$-$SiO_{2}$, and $TiO_{2}$-$Al_{2}O_{3}$) of the granulated type were also fabricated using combinations of free-standing oxide particles. The granulation of mixed oxides was obtained in a manner comparable to that used for single oxides, with the utmost attention given to obtaining the mutual suspension of the free-standing oxides in water via the use of PAA. Following the initial extrusion pelletizing process, the granulated particles were dispersed in LDPE through a film-blowing process.

It is noted that for both techniques, incorporating either free-standing or granulated particles, the extrusion process was identical, with the temperature kept at 250 °C, in order to ensure the stability of poly(acrylic acid) in the granulated samples [25]. The granulation technique described herein is a hybrid approach [18] between direct solvent blending [26,27,28] and direct melt blending [26,29,30]. A detailed report of the 19 films produced incorporating both techniques is given in Table 3.

### 2.4. Sample Characterization

The fabricated films were morphologically and optically characterized using a wide variety of techniques. The use of a Zeiss Axiolab 5 microscope (Zeiss, Oberkochen, Germany), in transmission mode, with an A-Plan, ×63, objective lens allowed for direct imaging of the films and particle dispersion in the matrix.

Roughness evaluation was achieved through AFM measurements using a Brüker Multimode device (VEECO Multimode, Santa Barbara, CA, USA), equipped with a Nanoscope III controller. The polymer film surface was investigated in intermittent (tapping) mode, under ambient conditions.

The chemical signature of the samples was investigated through Raman spectra, collected using an i-Raman Plus spectrometer (B&W TEK Inc., Plainsboro, NJ, USA). The excitation source was a diode laser (785 nm) with a maximum output power of 300 mW.

The chemical composition measurements were complemented using a theta–theta diffractometer (Rigaku Ultima IV, Tokyo, Japan) equipped with a Cu tube, operated at 40 kV and 40 mA, using a parallel X-ray beam (Cu$K_{\alpha }$, λ=0.1542 nm) conditioned by an X-ray mirror. The patterns were collected in Bragg–Brentano scans in the range of $10^{\circ }$–$60^{\circ }$ 2θ, with $0.05^{\circ }$ step and speed of $0.3^{\circ }$ 2θ/min.

Finally, UV-Vis-IR transmission and diffuse reflection spectra of the samples and reference material (spectolon) were recorded in the 250–2500 nm range using a Perkin Elmer Lambda 1050+ spectrophotometer (Perkin Elmer, Waltham, MA, USA), incorporating an integrated sphere setup (scan speed of 548 nm/min, slit width of 2.0 nm).

## 3. Results and Discussion

**Figure 1:**
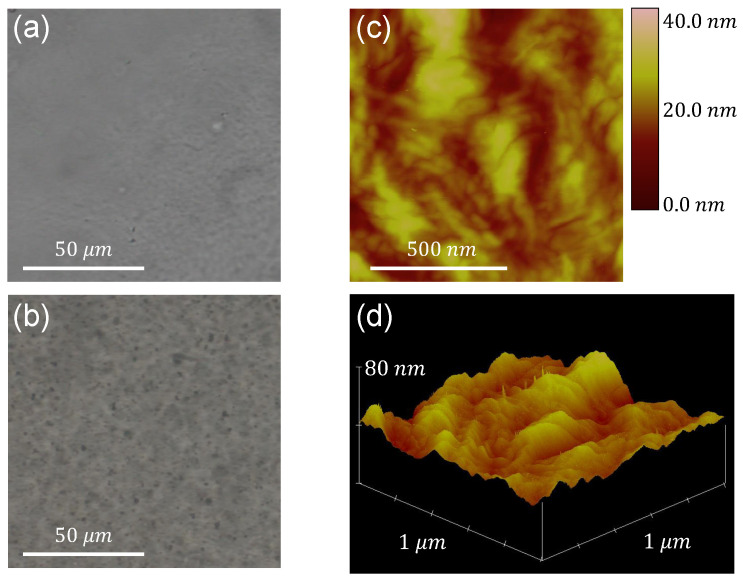
Optical microscope images of (**a**) neat LDPE and (**b**) the film containing ZnO (< 5 μm). (**c**) AFM image with colorscale indicating *z*-coordinate values and (**d**) 3D plot of *z*-coordinate for a surface of size 1×1 μm${}^{2}$, of the film containing ZnO (< 5 μm).

The image acquisition of metal oxides embedded in the films is done using the optical microscope, albeit only for the case of the larger particle sizes. In Figure 1a,b, the neat LDPE film and a film containing ZnO (< 5 μm) particles, respectively, are depicted, providing an initial assessment of the particles’ dispersion inside the polymer matrix.

The roughness of each film’s surface is subsequently characterized by means of AFM measurements. A phase image and a 3D plot of the *z*-coordinate of the film’s surface acquired through this technique are presented in Figure 1c,d, respectively, for the case of the aforementioned ZnO sample. Characterization of the roughness of the film’s surface is achieved by determining the Ra (arithmetic average) and RMS (root mean square) roughnesses. Both parameters are calculated using the measurements of the *z*-coordinate (height) of the surface as follows [31]:

(1) $$Ra=\frac{\Sigma_{i=1}^{N}\left|z_{i}\right|}{N},$$

(2) $$RMS=\sqrt{\frac{\Sigma_{i=1}^{N}z_{i}^{2}}{N}},$$

where $z_{i}$ is the distance from the average surface level, and *N* is the number of points. The roughness values are calculated for all samples, and it is concluded that the direct blending technique produces films with very similar surface morphology, regardless of the type and size of dispersoid embedded in the polymer. As an example, the values extracted for all ZnO-containing samples and neat LDPE, calculated from 1×1 μm${}^{2}$ images, are presented in Table 4.

**Figure 2:**
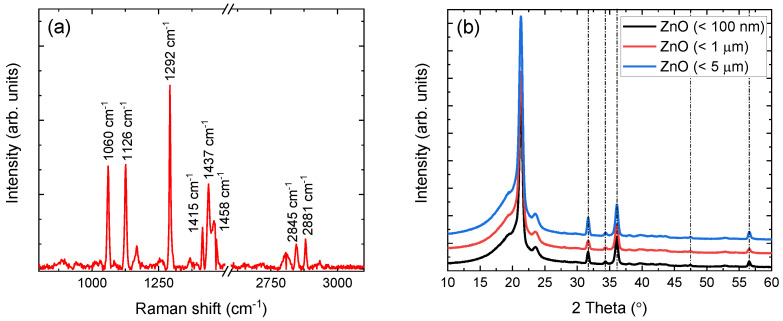
(**a**) Raman shift lines of LDPE in ZnO (< 5 μm) containing film, with an axis break for clarity. (**b**) XRD patterns of ZnO dispersoids embedded in LDPE. Nanoparticle sizes of < 100 nm (black), < 1 μm (red), and < 5 μm (blue) are shifted vertically for clarity.

Each sample chemical signature has been verified via complementary Raman and XRD techniques. Raman shift lines, in the range of 800–3100 cm${}^{-1}$, of the ZnO (< 5 μm) containing film are depicted in Figure 2a, after fluorescence background subtraction. The observed lines correspond to LDPE and are in very good agreement with the literature [32,33,34,35,36]. The 1060 cm${}^{-1}$ and 1126 cm${}^{-1}$ lines arise from vibrations of the asymmetric and symmetric -*C*-*C* stretching, respectively. The characteristic line at 1292 cm${}^{-1}$, corresponding to the -$CH_{2}$ twisting vibrational mode in the polyethylene crystalline phase, is also clearly resolved. Additional lines at 1415 cm${}^{-1}$, 1437 cm${}^{-1}$, and 1458 cm${}^{-1}$ are associated to one wagging and two scissoring modes of the -$CH_{2}$ groups, respectively. Finally, the two lines observed at 2845 cm${}^{-1}$ and 2873 cm${}^{-1}$ are attributed to $CH_{2}$ asymmetric and symmetric stretching, respectively.

Although the polymer percentage in each sample allows the direct observation of Raman shift lines, this is not the case for the dispersoid, whose percentage is significantly lower (i.e., 2%). For this reason, the XRD patterns of all the ZnO-containing films were acquired and are shown in Figure 2b. In addition to the characteristic peaks of LDPE, also presented in previous work [18], all samples yield the characteristic peaks of hexagonal ZnO at $31.77^{\circ }$, $34.43^{\circ }$, $36.25^{\circ }$, $47.55^{\circ }$, and $56.60^{\circ }$ 2θ [37]. The FWHM values of all ZnO peaks (excluding the peak at $36.25^{\circ }$, which overlaps with an LDPE peak), without any processing, lie in the range between $0.36^{\circ }$ and $0.42^{\circ }$ 2θ, indicating crystallite sizes between 20 nm and 26 nm according to the Scherrer formula [38]. This latter result, confirmed further by initial spectroscopic analysis, is currently under study and will be presented in future work.

**Figure 3:**
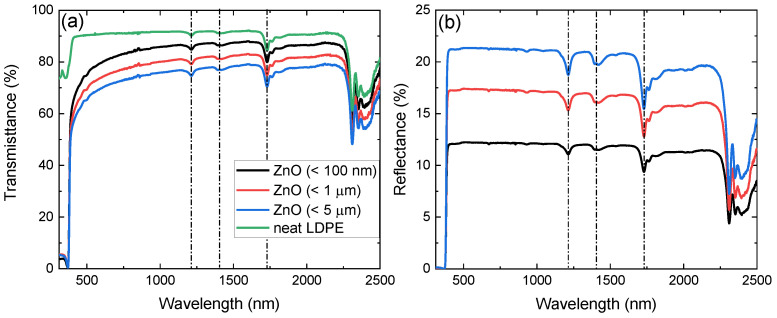
(**a**) Transmittance of neat LDPE (green) and ZnO of free-standing particle size of < 100 nm (black), < 1 μm (red), and < 5 μm (blue) embedded in LDPE from 310 nm to 2500 nm. (**b**) Reflectance of the latter with the exception of neat LDPE, omitted for clarity. The dash-dot lines in the spectra denote the absorption peaks of LDPE.

The experimentally acquired transmission and reflection spectra of the produced ZnO nanocomposite films are depicted in Figure 3a,b, respectively. Spectra below 310 nm are not presented due to low signal-to-noise ratio originating from high absorption. Neat LDPE spectra are also shown in Figure 3a, while they are omitted in Figure 3b for clarity. The characteristic absorption features of LDPE at 1192 nm, 1394 nm, and 1730 nm are present in both transmission and reflection spectra of the nanocomposite samples [39]. While neat LDPE exhibits a high transmission plateau, a clear decrease of the transmission signal is observed with increasing free-standing ZnO particle size embedded in LDPE. This is further confirmed through the reflection spectra, where for the dispersoids of 5 μm size, the lower transmittance and, correspondingly, higher reflectance is a direct result of their larger effective cross-section. Furthermore, with the exception of the ZnO band gap region becoming dominant below 400 nm, the spectra exhibit similar morphological characteristics with neat LDPE, as also pointed out in previous work [18]. It is noted that comparison between films containing free-standing $SiO_{2}$ and $Al_{2}O_{3}$ particles of different sizes yields extremely similar results.

**Figure 4:**
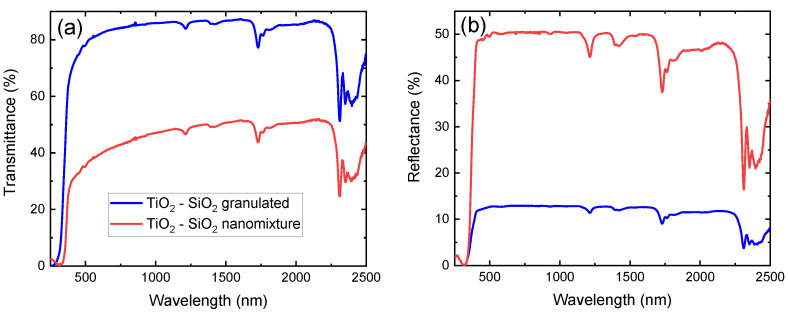
(**a**) Transmittance and (**b**) reflectance of $TiO_{2}$-$SiO_{2}$ nanoparticles of nanomixed (red) and granulated (blue) nature embedded in low-density polyethylene (LDPE) from 250 nm to 2500 nm.

Nanocomposite films of unary and mixed composition, fabricated with the use of both techniques, are here used for a direct comparison of their optical performance. In Figure 4a,b, the transmission and reflection spectra, respectively, of $TiO_{2}$-$SiO_{2}$-containing samples are presented in the 250 nm–2500 nm range. The absorption features of neat LDPE are in excellent agreement with previous works [18,39], while the interaction of the films with light is significantly stronger for the sample containing a nanomixture of free-standing particles. This is observed in the entire spectral range under investigation, with the notable exception of the band gap region. It is therefore concluded that the secondary particles have a significantly larger cross-section, and thus their dispersion should be significantly less homogeneous. The results are similar in the case of pure $TiO_{2}$ and $TiO_{2}$-$Al_{2}O_{3}$-containing films produced by both fabrication techniques.

**Figure 5:**
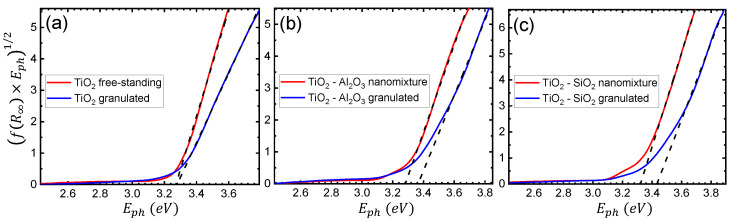
Tauc plots of the diffuse reflectance spectrum with linear fits (black dashed lines) for (**a**) $TiO_{2}$, (**b**) $TiO_{2}$-$Al_{2}O_{3}$, and (**c**) $TiO_{2}$-$SiO_{2}$ nanoparticles of both free-standing (nanomixture) and granulated nature. The intersection point of each linear fit with the horizontal axis corresponds to the extracted energy band gap $E_{g}$.

Further analysis focused on the theoretical extraction of the primary size of the particles in the samples produced by both techniques. The pseudoabsorbance was thus calculated through the Kubelka–Munk function $f(R_{\infty })$, using the following formula [40,41]:

(3) $$f\left(R_{\infty }\right)=\frac{{\left(1-R_{\infty }\right)}^{2}}{2R_{\infty }},$$

where $R_{\infty }=\frac{R_{sample}}{R_{spectolon}}$ and $R_{sample}$, $R_{spectolon}$ are the reflectance of the sample and reference material, respectively. The results presented in this case are based on higher resolution measurements of diffuse reflectance, in the 250–710 nm range, with a slit width of 0.2 nm. Tauc plots for the $TiO_{2}$, $TiO_{2}$-$Al_{2}O_{3}$, and $TiO_{2}$-$SiO_{2}$ samples, of both free-standing and granulated nanoparticles, are presented in Figure 5a–c, respectively. Following the indirect transition for anatase $TiO_{2}$, the Tauc formula here takes the following form [41]:

(4) $${\left(f\left(R_{\infty }\right)\cdot E_{ph}\right)}^{1/2}=A\left(E_{ph}-E_{g}\right),$$

where *A* is an energy-independent constant, and $E_{ph}$ and $E_{g}$ are the photon and band gap energies, respectively. Hence, the energy band gap of each material was estimated using the intersection value of the horizontal energy axis with the linear fit of the function of Equation (3) [41,42]. In the case of pure $TiO_{2}$, the extracted band gap is 3.26±0.02 eV and 3.28±0.01 eV for the free-standing and granulated cases, respectively, indicating similar results for both fabrication techniques. However, for the films containing free-standing and granulated $TiO_{2}$-$Al_{2}O_{3}$ particles, the calculation yields 3.29±0.01 eV and 3.34±0.03 eV, respectively. The observed difference is attributed to the smaller primary size of the granulated particles.

The difference is even more pronounced for $TiO_{2}$-$SiO_{2}$, where the band gaps were determined to be 3.32±0.01 eV and 3.43±0.03 eV for the free-standing and granulated cases, respectively. It is therefore concluded that, especially in the case of mixed oxides, the granulation process results in a significantly smaller primary size of the nanoparticles, due to the quantum size effect [43,44,45]. Furthermore, even though in both the free-standing and granulated cases, the addition of $Al_{2}O_{3}$ and $SiO_{2}$ leads to, correspondingly, a higher energy band gap with respect to pure $TiO_{2}$, the difference in the case of the granulated nanoparticles is more profound. A more efficient and controllable tuning of the end product’s optical properties is exhibited in the granulated case, due to the direct synergy of the two species. Such findings were verified by measurements on films containing $SiO_{2}$, $Al_{2}O_{3}$, and $SiO_{2}$-$Al_{2}O_{3}$ nanoparticles, omitted here for brevity.

The production of nanocomposite polymer films via the granulation technique entails that both single and mixed oxide granules are made up of a spatially random distribution of particles that adhere indiscriminately to each other by virtue of the poly(acrylic acid) that is adsorbed on their surface. Given that these granules are made up of particles that adhere to each other, once under the continuous exertion of uniform pressure, they start to disintegrate progressively into smaller fragments that are simultaneously and unrestrictedly dispersed into the polymeric matrix. As the extrusion process evolves, the fragmentation continues until all particles are released into the LDPE, preserving their primary size. Therefore, for a given composition and primary particle size of a dispersoid, the granulation technique establishes a standard for comparative evaluation from which the changes in optical performance can be measured, as both the primary and secondary particle sizes are diversified. In the case of free-standing particles, regulation of the dynamics (i.e., pressure, temperature, number of cycles) of the extrusion process, as well as a study focusing on the effect of the secondary size of the dispersoids on transmission and reflection spectra, are expected to lead to further optimization of their optical performance. Furthermore, complementary characterization techniques, such as spectroscopic ellipsometry [46,47] for the extraction of complex refractive index values, and photoluminescence [48] for the determination of sample aging, can lead to a more comprehensive mapping of their properties.

## 4. Conclusions

In this work, a comparative study has been conducted between nanocomposite polymer films containing unary and mixed oxide compositions produced by free-standing and granulated dispersoids, using the latter as a standard. The results show that the films produced using free-standing particles are less homogeneous due to the evidently larger cross-section of the dispersoid’s secondary size, as exhibited in optical transmission and reflection measurements for both unary and mixed compositions. Furthermore, the primary size of the dispersoids has been found to be larger, specifically in the case of mixed oxides, while a less direct interaction of the involved species is indicated through band gap energy extraction.

The critical achievement of the granulated method was the optical properties tailoring capability, due to the predetermined proportions of the mixed oxides, which were, in turn, characterized necessarily by a uniform secondary and primary particle size distribution. In the framework of this work, however, it is shown that the tailoring capability can be further enhanced by regulating the secondary size of free-standing oxide particles. This parameter provides complementary control of the optical properties both in the energy band gap region and, predominantly, in the VIS-IR range.

It is concluded that a tuning effect of the optical properties is feasible, albeit less controlled than in the standard granulated state, through this low-cost, free-standing particle technique. This can be proven sufficient on a per-application basis, such as optical coatings for glass surfaces and optical elements, optical filters, and sensors. 

## Figures and Tables

**Table 1:** Free-standing size of ZnO, $TiO_{2}$, $SiO_{2}$, and $Al_{2}O_{3}$ particles embedded in single or mixed formulation in LDPE.

Free-Standing Particle Size Range	ZnO	$TiO_{2}$	$SiO_{2}$	$Al_{2}O_{3}$
< 100 nm	< 100 nm	25 nm	12 nm	40–50 nm
0.1–1 μm	< 1 μm	-	0.5 μm	0.3–0.6 μm
1–10 μm	< 5 μm	-	-	< 10 μm

**Table 2:** Mass composition per 1–1 equimolar type of mixed oxide. Nanoparticle free-standing size is 12 nm, 40–50 nm, and 25 nm for $SiO_{2}$, $Al_{2}O_{3}$, and $TiO_{2}$, respectively.

Equimolar Mixtures	$SiO_{2}$ (g)	$Al_{2}O_{3}$ (g)	$TiO_{2}$ (g)
$TiO_{2}$-$SiO_{2}$	8.6	-	11.4
$Al_{2}O_{3}$-$SiO_{2}$	7.4	12.6	-
$TiO_{2}$-$Al_{2}O_{3}$	-	11.2	8.8

**Table 3:** Detailed account of nanocomposite films produced, indicating active material, particle size, and type of embedded dispersoid.

Sample	Oxide Type	Size	Technique
1	ZnO	< 100 nm	free-standing
2	ZnO	< 1 μm	
3	ZnO	< 5 μm	
4	$SiO_{2}$	12 nm	
5	$SiO_{2}$	0.5μm	
6	$TiO_{2}$	25 nm	
7	$Al_{2}O_{3}$	40–50 nm	
8	$Al_{2}O_{3}$	0.3–0.6 μm	
9	$Al_{2}O_{3}$	< 10 μm	
10	$TiO_{2}$-$SiO_{2}$	25 nm/12 nm	
11	$Al_{2}O_{3}$-$SiO_{2}$	40–50 nm/12 nm	
12	$TiO_{2}$-$Al_{2}O_{3}$	25 nm/40–50 nm	
13	$TiO_{2}$	25 nm	granulated
14	$SiO_{2}$	12 nm	
15	$Al_{2}O_{3}$	40–50 nm	
16	$TiO_{2}$-$SiO_{2}$	25 nm/12 nm	
17	$Al_{2}O_{3}$-$SiO_{2}$	40–50 nm/12 nm	
18	$TiO_{2}$-$Al_{2}O_{3}$	25 nm/40–50 nm	
19	neat LDPE	-	-

**Table 4:** Roughness values of ZnO composite films and neat LDPE.

	Ra (nm)	RMS (nm)
ZnO (< 100 nm)	3.5	4.5
ZnO (< 1 μm)	2.9	3.8
ZnO (< 5 μm)	3.4	4.3
neat LDPE	4.2	5.3

## Data Availability

The data presented in this study are available upon request from the corresponding authors.
